# BRIDGE EMPLOYMENT OR ENCORE CAREER? PREDICTORS THAT DISTINGUISH LATER LIFE CAREER TRANSITIONS

**DOI:** 10.1093/geroni/igad104.3205

**Published:** 2023-12-21

**Authors:** Yun taek Oh

**Affiliations:** University of Michigan, Ann Arbor, Michigan, United States

## Abstract

Moving away from the traditional lock-step retirement to multiple retirement pathways, later-life career transition has been prevalent for about a half-century (Ruhm, 1990). So-called “encore career,” many older workers start their second career after leaving their original career jobs. However, leaving one’s career job is not a distinct prequel to an encore career; instead, it is an essential step to bridge employment as well. While these two transitions have different purposes, reasons, and outcomes, the predictors distinguishing between them are relatively not well-documented. Using the sample of respondents with career jobs in the Health and Retirement Study, this study distinguished various later-life transitions by the voluntariness of career transition and the duration of labor force participation after the transition and then investigated the predictors that distinguish between bridge employment and encore career. Based on the theoretical predictions from multiple disciplines, such as the resource-based dynamic process model and continuity theory, I stated and tested a set of hypotheses using multinomial logit estimation. The result showed that financial, physical, and mental health resources, and retirement identity when transitioning to new jobs predict whether older workers take bridge employment or encore careers. This result provides a helpful hint to policymakers and employers who want to retain older workers in the labor force. Because the indicators for the voluntariness of career transition are available for those who left their career employers only, further research is needed once the voluntariness measures for other work adjustments are available.

